# Air Quality Over Major Cities of Saudi Arabia During Hajj Periods of 2019 and 2020

**DOI:** 10.1007/s41748-021-00202-z

**Published:** 2021-02-02

**Authors:** Ashraf Farahat, Akshansha Chauhan, Mohammed Al Otaibi, Ramesh P. Singh

**Affiliations:** 1 Department of Physics, College of General Studies, King Fahd University of Petroleum, and Minerals, Dhahran, 31261 Saudi Arabia; 2 Center for Space and Remote Sensing Research, National Central University, Taoyuan, Taiwan; 3 Health, Safety, Security and Environment Excellence Section, Industrial Security and Responsibility Care Department, Sahara International Petrochemical Company, SIPCHEM, Jubail Industrial City, 31961 Saudi Arabia; 4 School of Life and Environmental Sciences, Schmid College of Science and Technology, Chapman University, Orange, CA 92866 USA

**Keywords:** Air quality, Hajj 2020, COVID-19, Lockdown, Saudi Arabia

## Abstract

Mecca and Madinah are two holy cities where millions of people in general, visit throughout the years, during Hajj (Muslim's pilgrimage) time number of people visit these holy cities from different parts of the world is very high. However, the Government of Saudi Arabia only allowed 1000 pilgrims during the 2020 Hajj especially when the world is suffering from COVID-19. In the present paper, a detailed analysis of air quality parameters available from ground measurements have been carried over major cities of Saudi Arabia, Mecca, Madinah, and Jeddah from June to September 2019 and 2020. At Mecca and Jeddah, PM_10_, NO_2_ and CO concentrations are found to be higher in comparison to stations located close to Madinah. In addition, meteorological parameters, temperature, relative humidity, and wind directions are also analysed to understand changes associated with the meteorological parameters. Our detailed analysis shows a reduction in concentrations of pollutants during the 2020 Hajj, when the lockdown was observed in Saudi Arabia due to COVID-19. During June–August 2020 lockdown period, no major changes in PM_10_ concentration was observed since there were frequent dust events were observed. In contrast, our results show 44% reduction in NO_2_ (8.77 ppbv in 2019 to 4.97 ppbv in 2020) and 16% reduction in CO (0.85 ppm in 2019 to 0.72 ppm in 2020) concentrations during COVID-19 restrictions. The concentrations of NO_2_ and CO do not cause any issue for the air quality as NO_2_ and CO Indices lie within the green band (Air quality index 0–50). In Mecca, Madinah and Jeddah, the air quality is generally affected during Hajj, but during 2020, the concentration of particulate matter was influenced by local weather conditions.

## Introduction

Mecca and Madinah are the holy cities located in the western parts of the Kingdom of Saudi Arabia where millions of pilgrimage visits during Hajj (Muslim's pilgrimage) time. Over the years, the number of pilgrimages have increased up to three million (Ascoura 2013; Mirza 2020). The major parts of Saudi Arabia are occupied by desert, during the summer season with strong winds, desert soils uplifted in the atmosphere, cause heavy aerosols loading (Alam et al. 2014; Farahat et al. 2015; PME 2012). The desert areas of Saudi Arabia suffer from higher concentrations of particulate matter due to dust storms and construction activities (Alam et al. 2014; Farahat et al. 2016; Farahat 2019; Khodeir et al. 2012), with higher Ozone concentration and photochemical smog depending on the meteorological conditions (Lelieveld et al. 2009). Large number of vehicles on the road cause greenhouse gas emissions and volatile organic compounds (VOCs) (Rahman et al. 2017). Lim et al. (2018) suggested long-range transport of residual oil-burning aerosols that affect the air quality in Mecca even most of the oil deposits are located in the north-eastern parts of Saudi Arabia.

The COVID-19 affected many parts of the world, social distancing was observed, and work restrictions were imposed to mitigate the spread of the COVID-19. Such restrictions helped to improve the air quality of major cities of the world due to reductions in traffic and closure of industries (Chauhan and Singh 2020a,b; Connerton et al. 2020; Kumar et al. 2020; Singh and Chauhan 2020). The major anthropogenic activities related to the 2020 Hajj were also minimized that lead to reductions in the major pollutants in Mecca, Madinah, and Jeddah. In the present paper, we have carried out the analysis of air quality parameters (particulate matter and trace gases) in three major cities during 2019 and 2020 Hajj. Our results show pronounced changes in particulate matter, NO_2_ and CO concentrations.

### Major Cities of Western Saudi Arabia

**Fig. 1:**
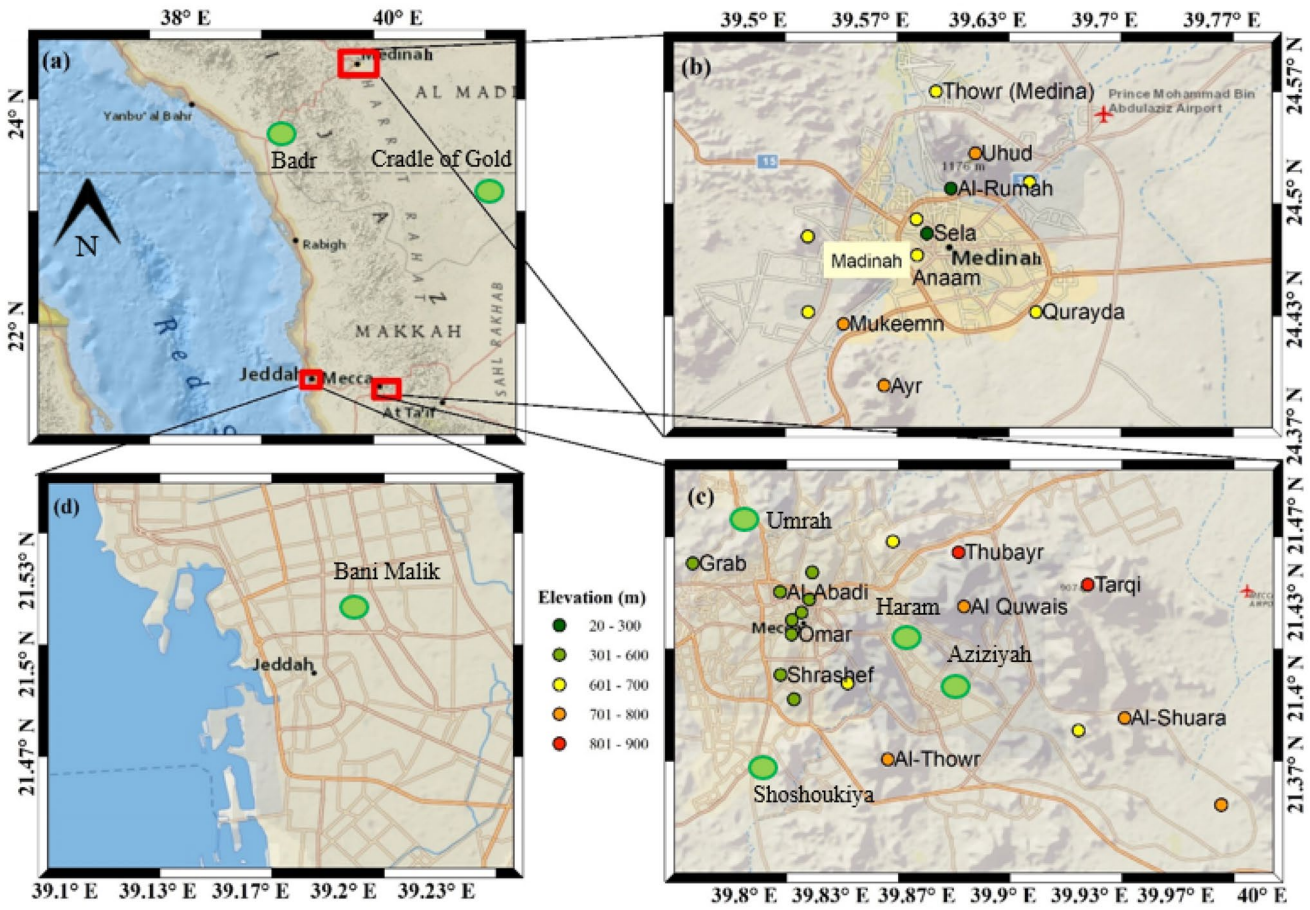
**a** The location of three city Mecca, Madinah, and Jeddah in Saudi Arabia (base layer source: National Geography). The colour circle shows the location of mountains peaks around, **b** Madinah, **c** Mecca and **d** Jeddah. The colour of each location depends on the elevation of the mountain peak. Location of ground station (shown by Green circles) for the measurement of various air quality parameters

Mecca, Madinah, and Jeddah are three major cities located at the western province of Saudi Arabia. Mecca is the holy city of the Islamic world, located in western Saudi Arabia (21.42° N, 39.82° E; population 1.96 million) inland from the Red Sea coast (United Nations 2018). The elevation of the city is 277 m above the mean sea level (amsl) and the city is surrounded by Sarawat Mountains from three sides (Fig. 1). Madinah (24.47° N, 39.60° E; population 1.3 million) in the northwest of Mecca and is the second holy city of the Islamic world. Jeddah (21.54° N, 39.17° E; population 3.2 million) is the second-largest populated cities after the capital city Riyadh. Mecca, Madinah, and Jeddah are located in the semi-arid/arid regions of Saudi Arabia with an average rainfall less than 130 mm, mainly during the winter season (Farahat 2016). During summer, the average maximum temperature often exceeds 38 ºC with lower precipitation (less than 10 mm) (Hasanean and Almazroui 2015; Howarth et al. 2020; Nayebare et al. 2018). Anthropogenic and natural emissions from dust storms, large construction activties, traffic, petrochemical, and cement industries affect particulate matter loading in the atmosphere (Al-Jeelani 2009; Othman et al. 2010; Seroji 2011; Mohammed et al. 2015, 2016; Munir et al. 2016; Lim et al. 2018; Nayebare et al. 2018; Al Otaibi et al. 2019) .

During 2019 Hajj time, around 2.5 million pilgrims gathered in Mecca (Mirza 2020). Each year, Hajj starts in Dhu al-Hijjah, in the last month of the Islamic Calendar, for 5-days duration. Pilgrims arrive to the city of Mecca about 5 days before the Hajj starts and leave 4–5 days after the last day of Hajj.

During Hajj, pilgrims visit from every country in the world, but the number of visitors within Saudi Arabia are the highest. As there is no airport in Mecca, many international pilgrims travel by air to King Abdulaziz International Airport (JED) in the city of Jeddah (about 85 km from Mecca) and domestic pilgrims travel to Mecca by local transport buses, cars, and taxis. In 2019, more than 18,000 buses were used to transport the pilgrims to Mecca (Vincent 2019). Some pilgrims come to the Prince Mohammad Bin Abdul-Aziz International Airport (MED) in the city of Madinah (about 450 km from Mecca) and Taif International Airport (TIF) (about 86 km) and use local transport to reach at the Hajj location.

The number of visitors during Hajj time is growing every year; as a result, human activities and traffic have also increased, affecting air quality (Siddique 2016; Simpson et al. 2014). Moreover, Mecca and Madinah are expanding at a rapid rate and large scale of construction and demolition activties, which is also affecting the air quality of the two holy cities (Abdel Hameed et al. 2016; Al-Jeelani 2009; Mohammed et al. 2015; Munir et al. 2016; Othman et al. 2010; Seroji 2011). The city of Jeddah has poor air quality mostly affected by desert dust, vehicular emission, and other anthropogenic activities (Barletta et al. 2017; Hussain et al. 2014; Mashat et al. 2018; Munir et al. 2013, 2016; Nayebare et al. 2018; Taylan 2013).

### COVID-19 and Hajj 2020

**Fig. 2:**
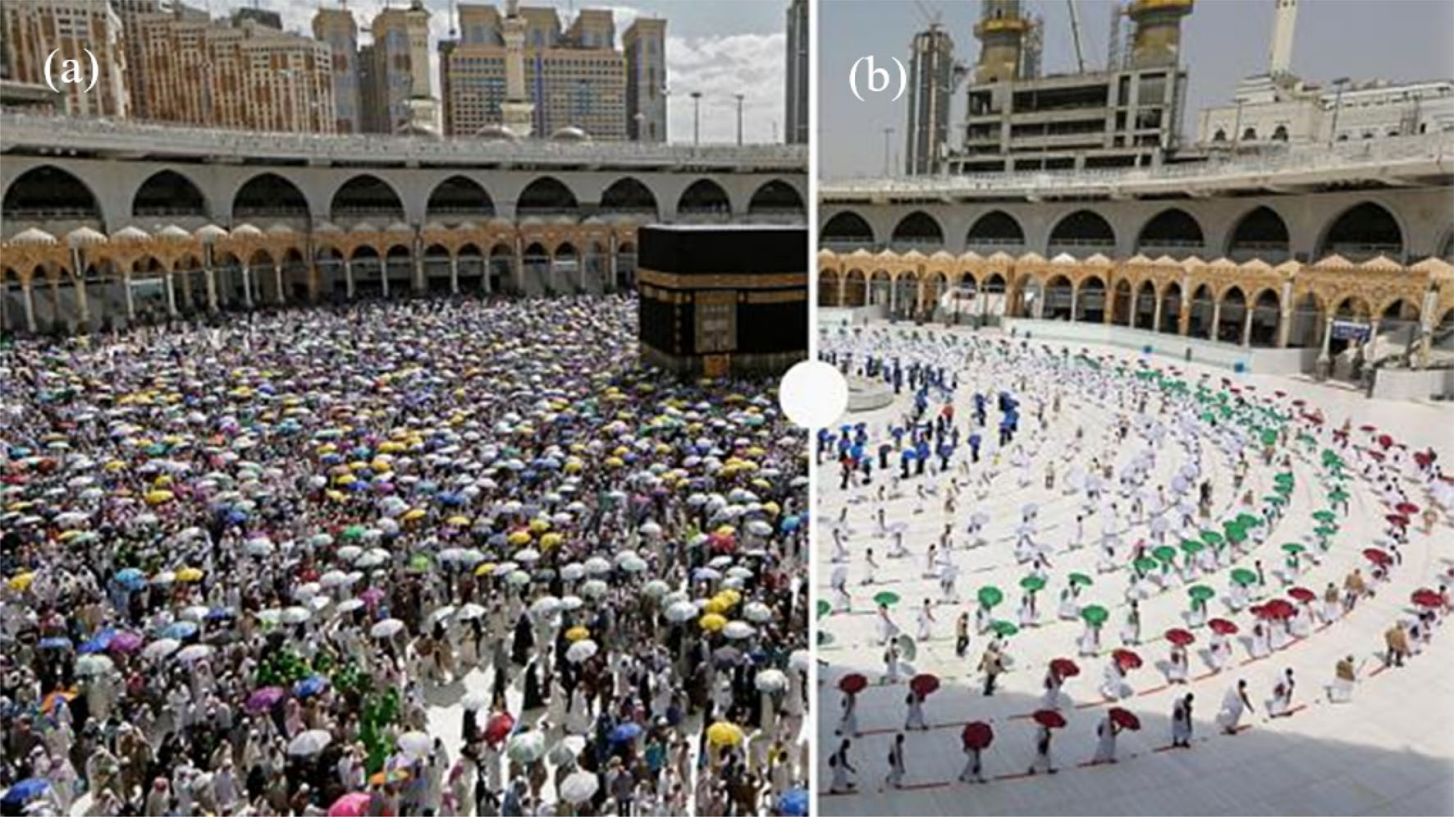
Pilgrims for Hajj in Mecca in **a** 2019 and **b** 2020 (Source: Saudi Ministry of Media/AFP and Euro News 2020)

In 2020, during Cthe OVID-19 pandemic, the Government of Saudi Arabia imposed a lockdown to stop the spread of COVID-19, however, more than 2, 87,262 people suffered in Saudi Arabia and more than 3000 people died until 31 August 2020 (Corona Tracker 2020). Hajj was also affected by the COVID-19, only the Government restricted 1000 pilgrims (SPA 2020) to Mecca (Fig. 2). Further, all pilgrims were restricted to stay in Mecca only and no one could travel to Madinah. Fourteen days of quarantine were imposed on pilgrims before and after the Hajj. Regular health check-ups, social distancing and electronic monitoring of everyone were mandatory during the 2020 Hajj period (Ministry of Hajj and Umrah 2020).

## Data Used and Methodology

The Hajj was organised during 9–14 August 2019 and 28 July–2 August 2020, respectively. We have considered three periods, before, during, and after for 2019 and 2020 Hajj periods. For each year, we have considered 10 days before and 10 days after the date of starting and end of Hajj. The air quality parameters (PM_10_, NO_2_ and CO) from ground stations located in Mecca, Madinah, and Jeddah are considered (Fig. 1).

### Air Quality Parameters

The air quality parameters for Mecca, Madinah, and Jeddah are taken from the World Air Quality Index Project (WAQIP 2020). Using standard monitoring equipment, the concentration of various pollutants is observed at all locations of the World Air Quality Index Project by each countries’ respective Environmental Protection Agency (EPA). In Saudi Arabia, the concentration of the various pollutants is provided by Saudi Arabia General Authority for Meteorology and Environmental Protection (MEWA 2020). The index of each parameter is calculated by WAQIP using the US Environmental Protection Agency formula (USEPA 2020). The raw data of PM_10_, NO_2_, and CO is not available through the website, however, the index of PM_10_, NO_2_, and CO are available through the WAQIP server. The list of all World Air Quality Index Project (WAQIP) stations are available from (WAQIP 2020).

We have considered air quality data for the periods July 2019–August 2020 from 7 ground stations (coordinates of these stations are given in Table 1), stations 1–4 are located in Mecca city, station 5 is located in Jeddah and Stations 6 and 7 are nearby to the Madinah (Fig. 1). The distance of station 6 is 70 km and station 7 is 100 km from the Madinah city center, no station is located in Madinah city.

**Table 1:** Name of stations and coordinates

SI. no.	City	Station name	Latitude (°N)	Longitude (°E)
1	Mecca	Aziziyah	21.40	39.88
2	Mecca	Umrah	21.51	39.79
3	Mecca	Al Shoqiyah	21.37	39.81
4	Mecca	Haram	21.42	39.83
5	Jeddah	Bani Malik	21.52	39.20
6	Madinah	Badr	23.78	38.80
7	Madinah	Cradle of Gold	23.50	40.89

In our analysis, we have also calculated the average concentration of each parameter using USEPA procedures and the details are given in USEPA technical report (USEPA 2020).

### Meteorological Parameters

We have carried out the analysis of meteorological parameters (air temperature, air pressure, relative humidity, wind velocity, and wind direction) for the periods 2019 and 2020 using the data provided by Reliable Prognosis. The website has been designed and supported by Raspisaniye Pogodi Ltd., St. Petersburg, Russia, since 2004. This website provides actual and forecast data of weather using various ground observations. The forecasts are prepared by the Met Office, the United Kingdom, and available through the website under the contract between the Met Office and Raspisaniye Pogodi Ltd. Information on the actual weather comes from the server of international data exchange, NOAA, United States, in SYNOP and METAR formats. All the data are fully quality controlled and are freely available.

## Result and Discussion

### Meteorology of Mecca (Jeddah) and Madinah

**Fig. 3:**
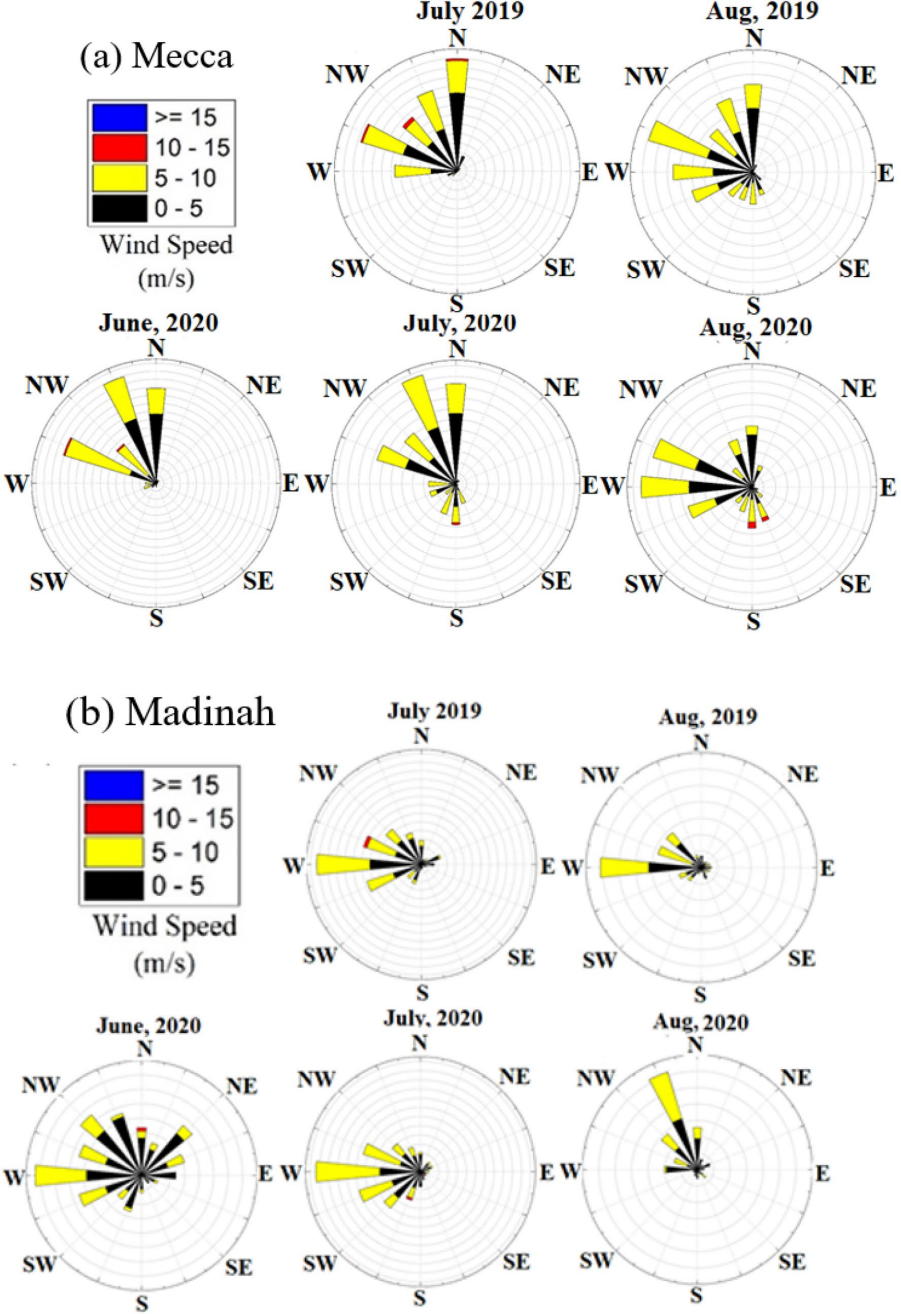
The wind rose diagrams for **a** Mecca and **b** Madinah during June, July and August 2019 and 2020. The dominant wind at Mecca is north-west and westerly winds during July 2019 and 2020. At Madinah, dominant westerly winds were observed during 2019 and 2020. The wind direction at Jeddah and Mecca are observed to be similar (Weather in the World 2020)

In Mecca and Jeddah, dominant wind is northerly and westerly during July and August 2019, with a small component of southerly wind during August 2019 (Fig. 3). In June 2020, the dominant wind is north-westerly to westerly. During July 2020, wind is westerly with a southerly component and in August 2020, the wind pattern is north-westerly to westerly, similar wind pattern was observed in these months during 2019 (Fig. 3).

In Madinah, dominant wind is south-westerly to northerly during June, July and August 2019 and 2020, almost similar for these months in the year 2019 without major changes in wind direction.

We have also analysed air temperature, relative humidity, and air pressure at Mecca, Madinah, and Jeddah. The meteorological parameters available of Mecca and Jeddah are the same as assimilated data of various resources provided by Reliable Prognosis, so we have shown only for Mecca.

**Fig. 4:**
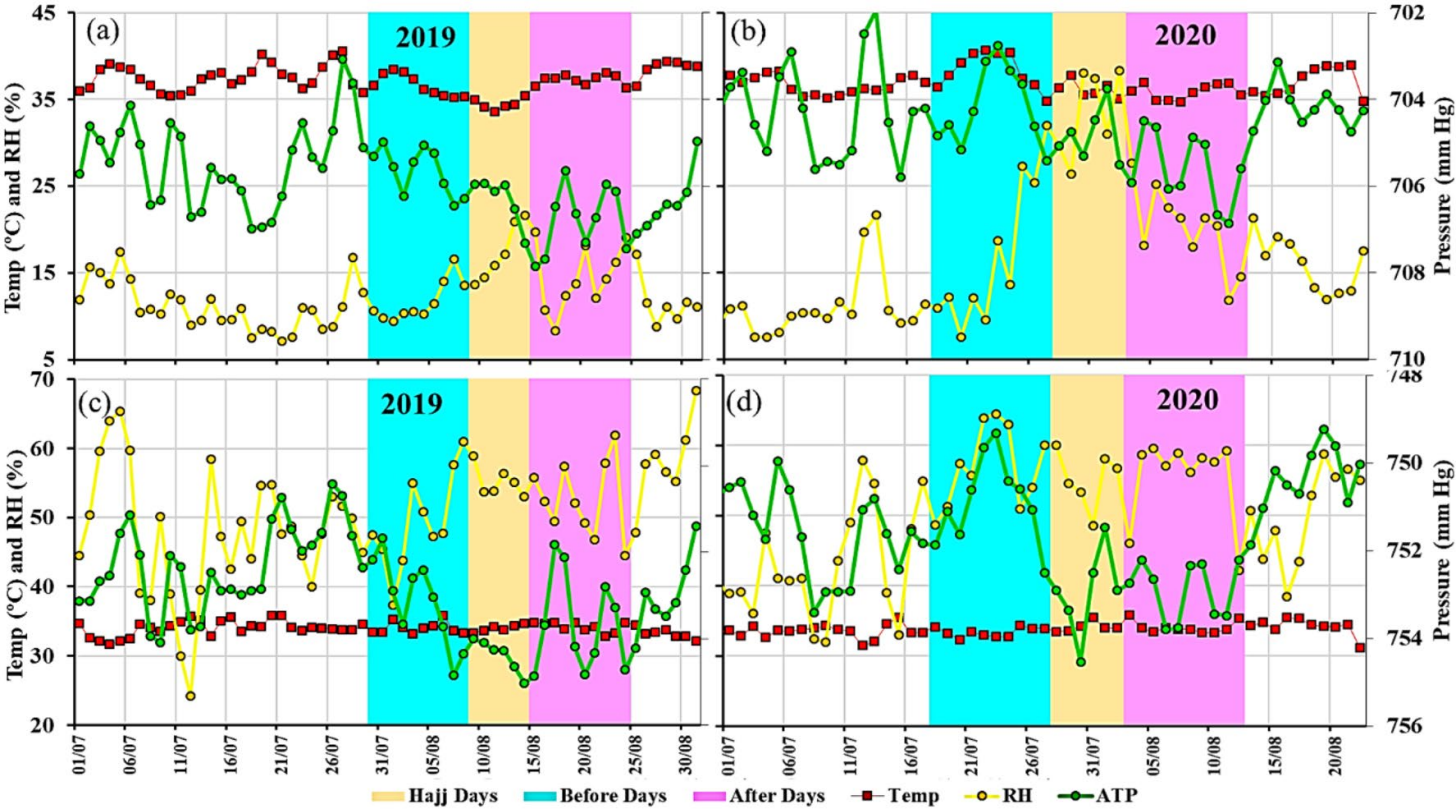
Temporal variations of air temperature (ºC), relative humidity (%) and air pressure (mm Hg) at **a** Madinah during 2019; **b** Madinah during 2020; **c** Mecca during 2019 and **d** Mecca during 2020

In Mecca, the average temperature increases during June–August (Fig. 4); the mean temperature during July and August 2019 was 34 ºC. During 2020, the average temperature varies, 32 ºC during June, 33.5 ºC during July and 34 ºC during August, the air temperature in both years was similar during Hajj periods. A reduction in 0.17% is observed in air pressure and 1.80% rise in average relative humidity during July and August 2020 compared to 2019 in Mecca. In Madinah, fall of about 1.05% in air temperature and 0.18% in air pressure was observed, whereas, 40% rise in relative humidity was observed in Madinah during 2020. A sudden rise in relative humidity was observed during the 2020 Hajj in Madinah and a similar rise was observed during the 2019 Hajj.

### Variations in Particulate Matter concentrations

**Fig. 5:**
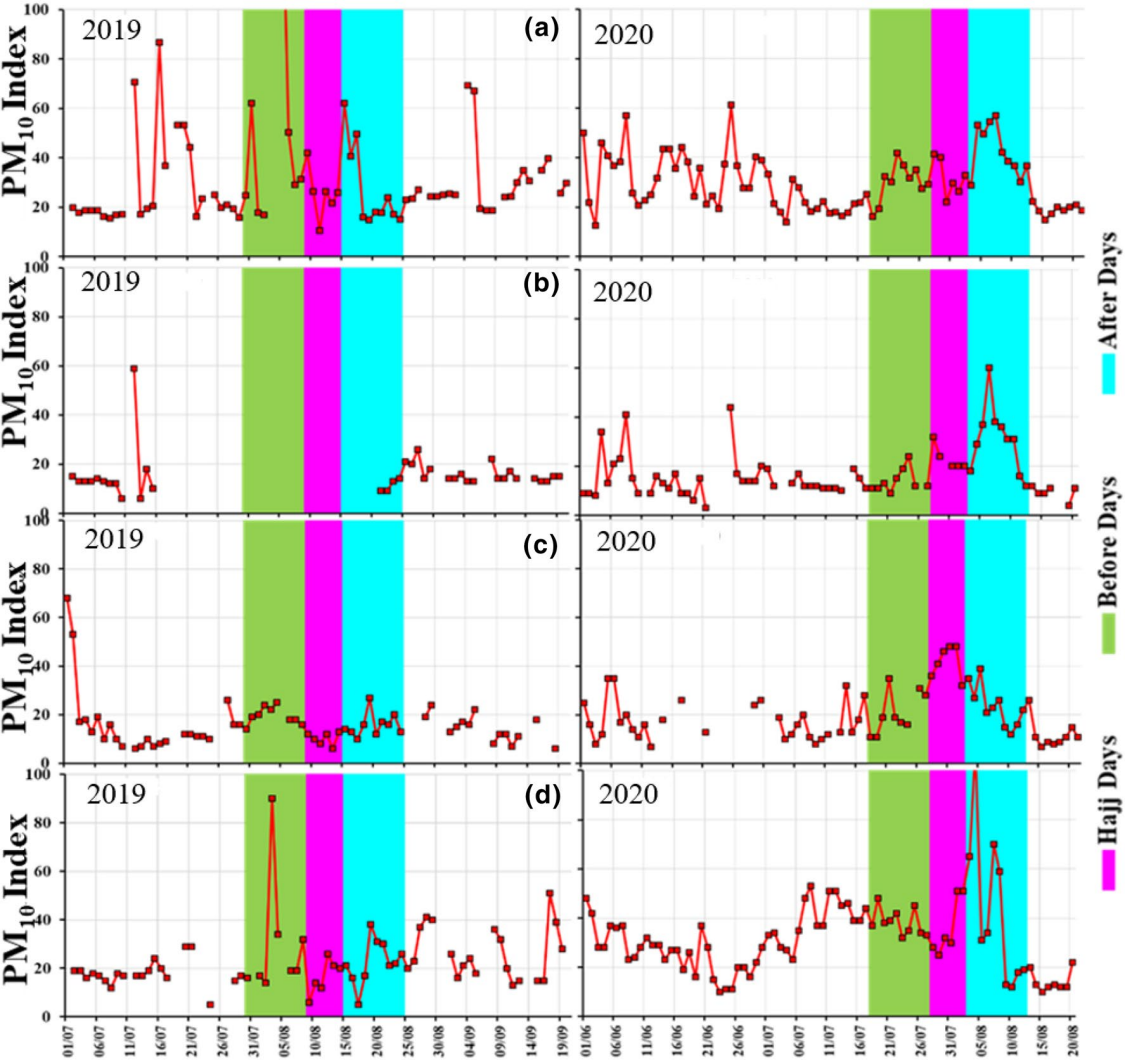
Temporal Variations of PM_10_ index over **a** Mecca, **b** Jeddah, **c** Cradle of Gold Station, Madinah and **d** Badr Station, Madinah during 2019 and 2020, data is available for July 2019

Figure 5 and Table 2 show the temporal variations of PM_10_ index at Mecca, Jeddah, and Madinah.

**Table 2:** Average concentration of PM_10_, NO_2_ and CO at Mecca, Jeddah, COD and Badr stations

	Mecca		Jeddah		Near Madinah				
					Cradle of Gold			Badr	
	2019	2020	2019	2020	2019	2020	2019		2020
PM_10_ (µg/m^3^)									
Before	70.77	32.52	No data	15.12	21.12	22.44	32.54		41.36
During	27.45	34.65	No data	25.06	10.98	45.18	17.82		39.06
After	29.65	46.16	12.15	33.26	17.06	25.49	24.52		46.44
NO_2_ (ppbv)									
Before	9.60	5.30	No data	5.08	4.08	1.51	No data available		2.92
During	9.95	4.50	No data	2.16	4.86	1.44	7.56		1.98
After	9.86	3.48	11.23	2.38	4.86	1.08	3.24		3.02
CO (ppm)									
Before	0.83	0.71	No data	0.64	0.87	0.57	1.15		0.79
During	1.28	0.81	No data	0.48	0.92	0.81	0.79		0.70
After	1.08	0.75	2.06	0.86	0.92	0.90	0.99		0.66

In Mecca (Fig. 5a), during 2019, we found that from 30 July–08 August (before Hajj), the average PM_10_ index is 58.81 (equivalent to PM_10_ concentration 70.77 µg/m^3^). From 9 to 14 August 2019 (during Hajj), the average PM_10_ index was 25.42 (equivalent to PM_10_ concentration 27.45 µg/m^3^). From 15 to 24 August 2019 (after Hajj), the average PM_10_ index was 27.45 (equivalent to PM_10_ concentration 29.65 µg/m^3^). During 2020, we observed that during 18–27 July (before Hajj) the average PM_10_ index was 30.11 (equivalent to PM_10_ concentration 32.52 µg/m^3^). During 28 July–02 August (during 2020 Hajj), the average PM_10_ index was 32.08 (equivalent to PM_10_ concentration 34.65 µg/m^3^). During 3–12 August 2020 (after Hajj), the average PM_10_ index was 42.74 (equivalent to PM10 concentration 46.16 µg/m^3^).

Figure 5a shows that there was an increase in PM_10_ concentration before 2019 and 2020 Hajj. This increase was attributed to large anthropogenic emissions associated with traffic and human activities especially when pilgrims moved to Mecca. In 2020, PM_10_ concentration decreased by about 57.7% compared to 2019 before the Hajj period. PM_10_ concentration during the 2019 and 2020 Hajj was very comparable. This was due to the pilgrims stayed inside the holy sites in Mecca and transported by buses and trains, where small and private vehicles were restricted. This clearly shows that PM_10_ loading was mostly affected by a large number of vehicles used to transport pilgrims to Mecca and not necessarily affected by the number of pilgrims visiting the city. It was also interesting to observe that after Hajj, PM_10_ concentration increased by only 11.1% and 31.1% during 2019 and 2020 Hajj respectively. This was due to a number of pilgrims who stayed for some time in Mecca and also some of them visited other surrounding cities, like Madinah after Hajj. But prior to the Hajj periods, especially one or two days, the traffic was crowded which was seen from the poor air quality, PM_10_ concentration prior and after the 2020 Hajj was slightly higher compared to 2019, but this was attributed to strong wind and natural aerosol loading in the atmosphere (Fig. 3).

In Jeddah (Fig. 5b, Table 2), during 2020 Hajj, the average PM_10_ index was 11.25 (equivalent to 12.15 µg/m^3^) after Hajj while data is missing before and during the 2019 Hajj. In 2020, the average PM_10_ index was 14.00, 23.00 and 30.80 (equivalent to 15.12, 25.06 and 33.26 µg/m^3^), respectively, before, during and after Hajj. These are higher values compared with the average monthly values during 2019. Although the main airport (King Abdulaziz International) is located in Jeddah city where most of the pilgrims commute to Mecca and Hajj activities did not affect air quality in Jeddah during 2020. As data is missing for 2019, it was difficult to conclude if Jeddah had similar PM_10_ variations during the 2019 Hajj.

Near Madinah, in Cradle of Gold station, in 2019, the average PM_10_ index is 19.56, 10.17, and 15.80 (equivalent to 21.12, 10.98 and 17.06 µg/m^3^) before, during, and after Hajj, respectively. In 2020, the average PM_10_ index is 20.78, 41.83 and 23.60 (equivalent to 22.44, 45.18 and 25.49 µg/m^3^) before, during and after Hajj, respectively (Fig. 5c). These are higher values compared with the average monthly values during 2019. At Badr station (Fig. 5d), in 2019, the average PM_10_ index was 30.13, 16.50 and 22.70 (equivalent to 32.54, 17.82 and 24.52 µg/m^3^) before, during, and after Hajj, respectively. In 2020, the average PM_10_ index was 38.30, 36.17 and 43.00 (equivalent to 41.36, 39.06 and 46.44 µg/m^3^) before, during and after Hajj, respectively (Fig. 5d).

**Fig. 6:**
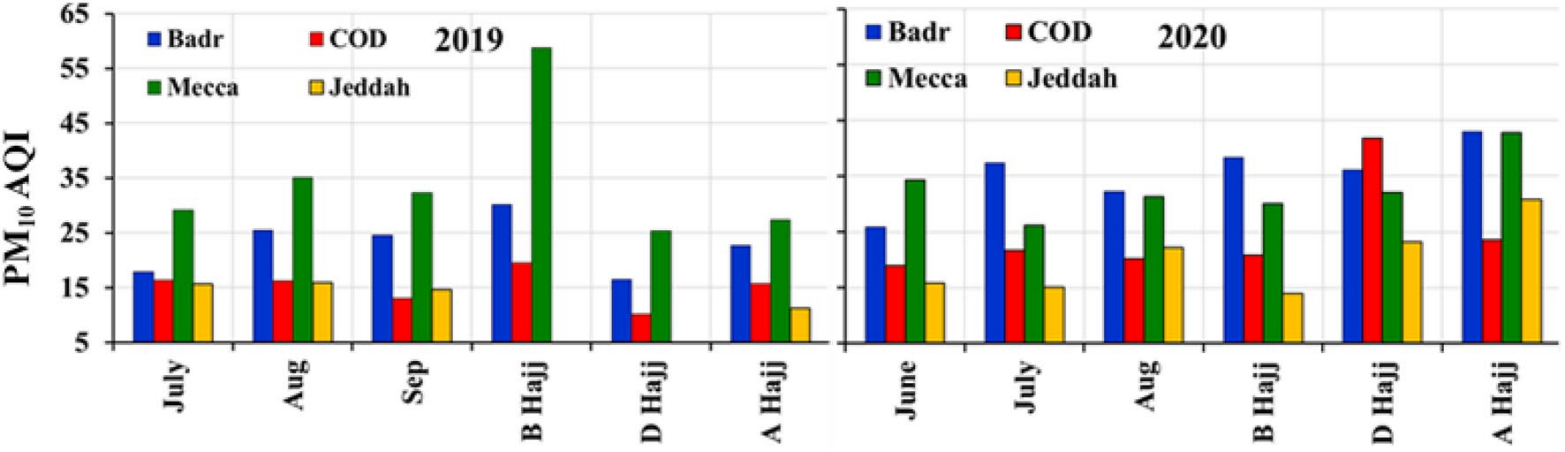
Average PM_10_ index over Jeddah, Badr Station Madinah, COD (cradle of gold) Station, Madinah, and Mecca during 2019 and 2020. **B** Hajj (before Hajj); **D** Hajj (during Hajj) and **A** Hajj (after Hajj)

Figure 6 shows the monthly average variations of PM_10_ index at Mecca, Jeddah, COD (Cradle of Gold) and Badr stations near Madinah. During 2019, we found an average PM_10_ index in Mecca was 59% higher compared to Jeddah, COD, and Badr, whereas, in 2020, PM_10_ index was found to be lower compared to COD and Badr. In 2020, pilgrims were restricted to visit Madinah, thus the observed PM_10_ concertation observed at COD and Badr stations were due to natural and local anthropogenic emissions.

**Fig. 7:**
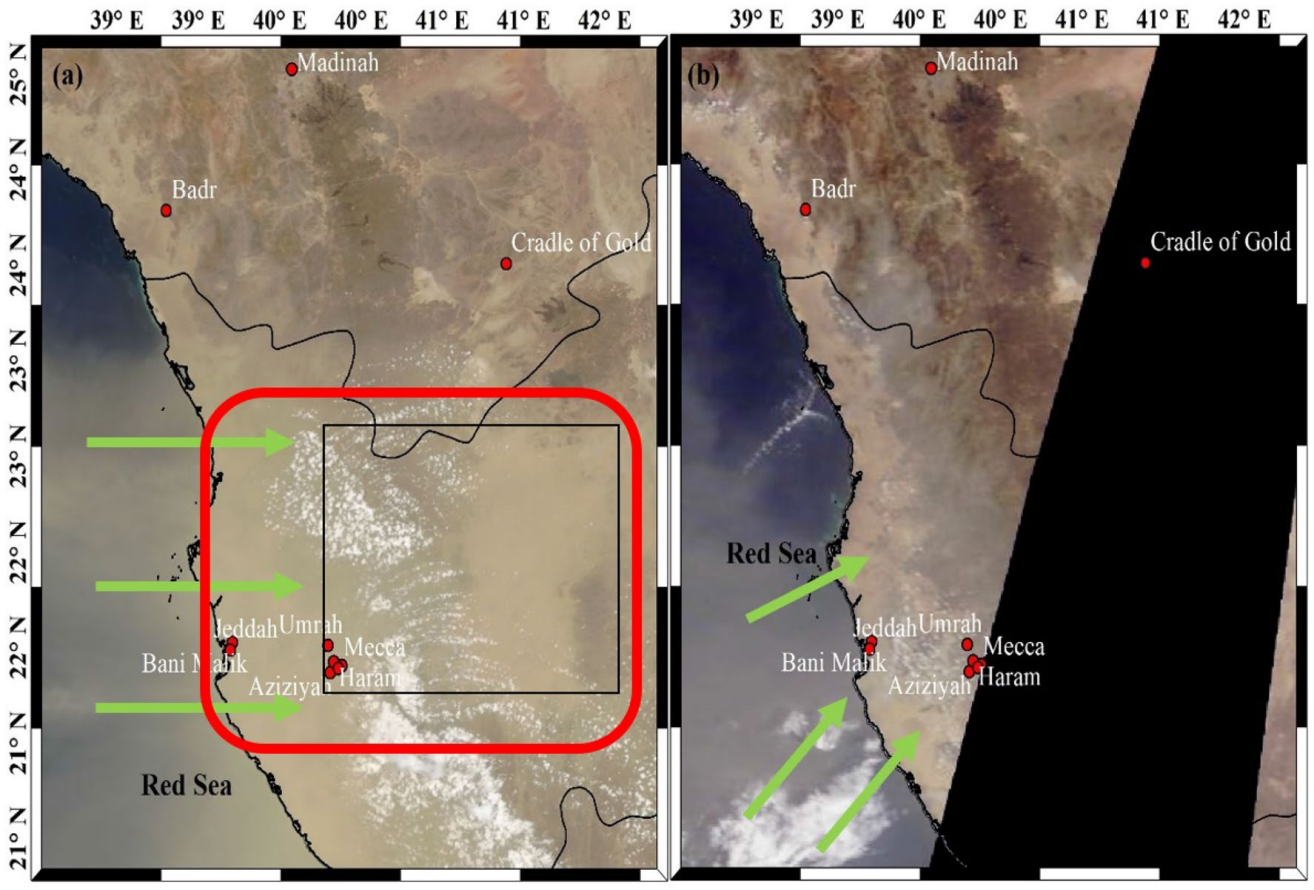
Terra MODIS true colour image (RGB) (Source of image: NASA World View 2020) of Western Saudi Arabia on **a** 06–08-2019 and **b** 05–08-2020 clearly showing dust over Red sea and Mecca District. The rectangular area with red and black color show dust-covered area, the green arrows show the movement of dust from the Red sea over land areas. Location of various stations are shown with red circles

We also observed a dust storm in Mecca and surrounding regions during 5–8 August 2019 in the Arabian Peninsula that enhanced the PM_10_ index more than 150 (equivalent to PM_10_ concentration 251 µg/m^3^) in Mecca (Fig. 7a). In 2020, we found a sudden rise in PM_10_ Index (concentration) during 3–12 August at Mecca, Badr and Jeddah. Further analysis shows that the region is continuously affected by the dust events (Fig. 7b), as a result the PM_10_ index enhanced and its affect is seen through the poor visibility. This indicates that dust storms and other meteorological conditions in Mecca and in Jeddah and Madinah (Badr and COD stations) are contributing to variations of PM_10_ concentration over the three cities.

### Changes in NO_2_ Concentrations

**Fig. 8:**
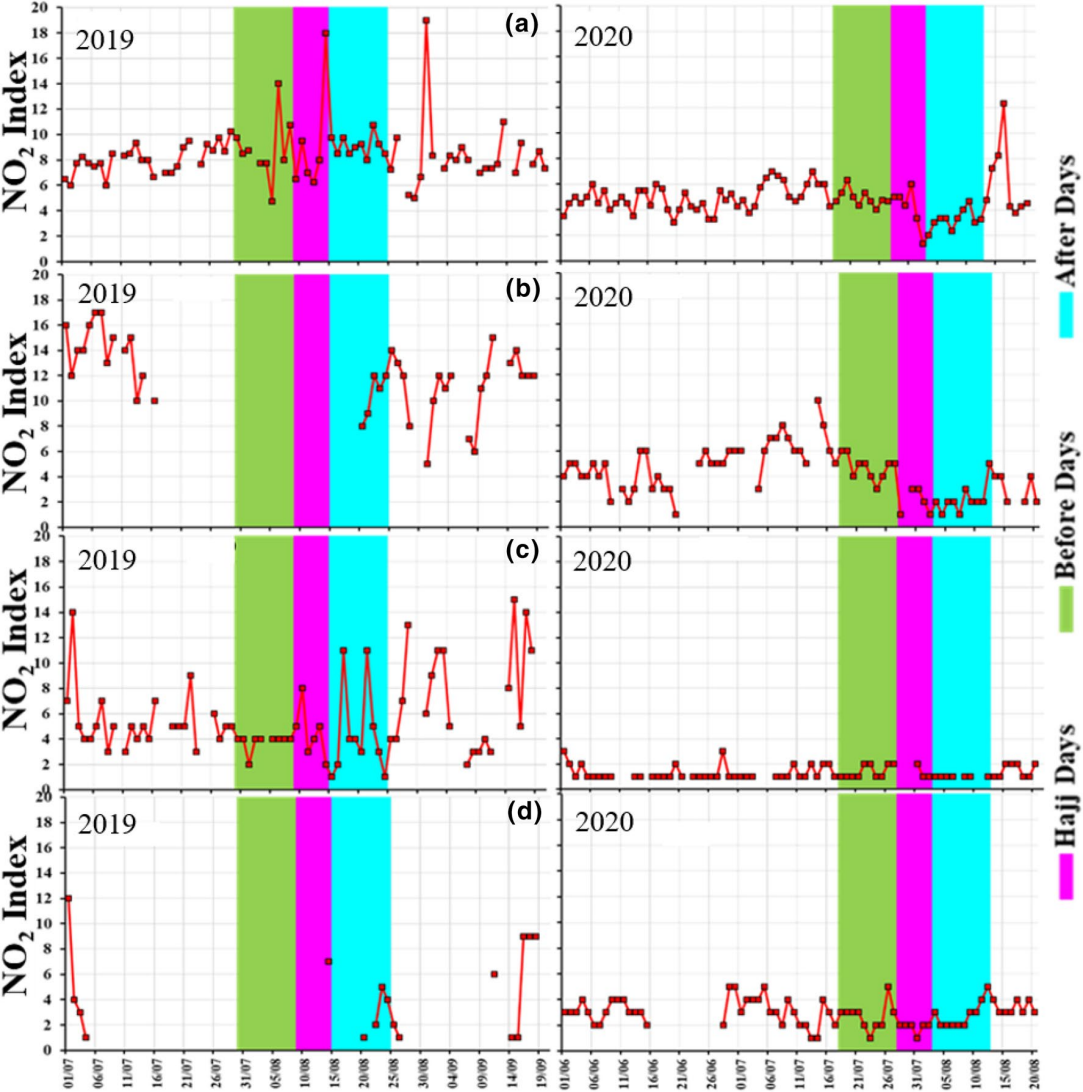
Temporal Variations of surface NO_2_ over **a** Mecca, **b** Jeddah, **c** Cradle of Gold Station, Madinah and **d** Badr Station, Madinah during 2019 and 2020

Vehicular emissions are considered one the major sources of surface NO_2_ in Saudi Arabia (Al Otaibi 2019, Farahat 2016). The temporal variations of NO_2_ are displayed over Mecca, Jeddah, COD and Badr stations near Madinah (Fig. 8, Table 2).

In Mecca, in 2019, the average NO_2_ Index is 8.89, 9.21, and 9.13 (equivalent to 9.60, 9.95 and 9.86 ppbv (parts per billion) concentration) before, during and after Hajj, respectively (Fig. 8a, Table 2). In 2020, the average NO_2_ Index is 4.91, 4.17 and 3.23 (equivalent to 5.30, 4.50 and 3.48 ppbv) before, during, and after Hajj, respectively. A reduction of 44% was found in the monthly mean NO_2_ in 2020. Interestingly, in 2019, a sharp increase in NO_2_ Index is observed before and after Hajj. This was clearly observed by the two peaks in NO_2_ index around 4 and 31 August (Fig. 8a). Similar increasing trend of NO_2_ is observed in 2020 but with low values compared to 2019. The increase is observed around 15 August, about 2 days after Hajj officially ended in 2020, which clearly shows the effect of traffic in increasing NO_2_ during 2019 Hajj compared to 2020 Hajj. We found an average reduction of 44% in the months of July and August 2020 and a reduction of more than 55% during the whole 2020 Hajj season in Mecca compared to 2019.

In Jeddah, in 2019, the average NO_2_ Index was 10.40 (equivalent to 11.23 ppbv) after Hajj, the station data of before and during Hajj in 2019 at Jeddah was not available. Enhancement in NO_2_ concentration was observed during 20–25 August 2019. In 2020, the average NO_2_ Index is 4.70, 2.0 and 2.20 (equivalent to 5.08, 2.16 and 2.38 ppbv) before, during and after Hajj, respectively (Fig. 8b). But we observed a reduction up to 62% in the month of July and 77% in the month of August 2020 compared to July and August during 2019.

Near Madinah, at COD station, in 2019, the average NO_2_ Index is 3.78, 4.50 and 4.50 (equivalent 4.08, 4.86 and 4.86 ppbv) before, during and after Hajj, respectively. In 2020, the average NO_2_ Index was 1.40, 1.33 and 1.00 (equivalent to 1.51, 1.44 and 1.08 ppbv) before, during and after Hajj, respectively (Fig. 8c). We observed a sudden enhancement in NO_2_ index after the 2019 Hajj, which could be attributed to pilgrims crowds towards Madinah after the Hajj as the COD station is located in the east of the Makkah /Al- Medina highway, which was used to reach Madinah from Mecca. During 2020 Hajj, a reduction in NO_2_ Index up to 70% was observed at COD station compared to 2019 Hajj.

**Fig. 9:**
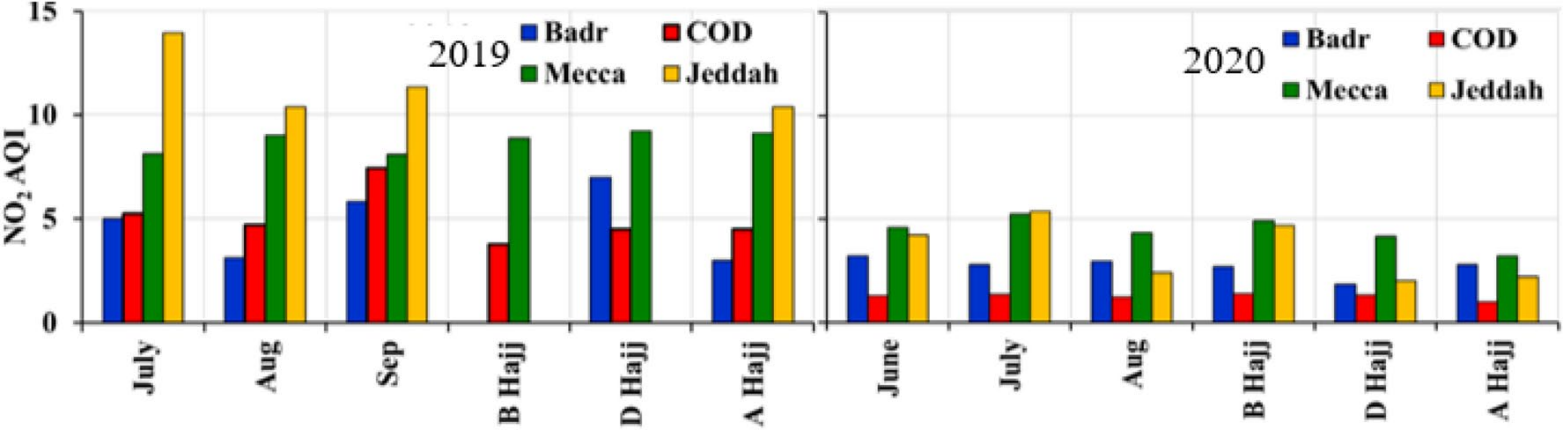
Average NO_2_ Index over Jeddah, Badr Station Madinah, COD (Cradle of Gold) Station, Madinah, and Mecca during 2019 and 2020. **B** Hajj (before Hajj); **D** Hajj (during Hajj) and **A** Hajj (after Hajj)

At Badr station, Madinah, only a few data points were available during the 2019 Hajj, so it was difficult to compare for two Hajj periods. At Badr station in 2019, the average NO_2_ Index was 7.00 and 3.00 (equivalent to 7.36 and 3.24 ppbv) during and after Hajj, respectively, and data was missing for before Hajj period. In 2020, the average NO_2_ Index was 2.70, 1.83 and 2.80 (equivalent to 2.9, 1.9 and 3.0 ppbv) before, during and after Hajj, respectively (Fig. 8d). In 2020, the average concentration was 53.6% lower at Badr station with respect to 2019. Here, we observed sudden enhancement (79.8%) just before the Hajj and 7 days after the end of Hajj (7.2%). An increase in traffic during Hajj, an enhancement in NO_2_ concentration was observed which remained within the healthy AQI limits (Fig. 9).

### Changes in CO Concentrations

**Fig. 10:**
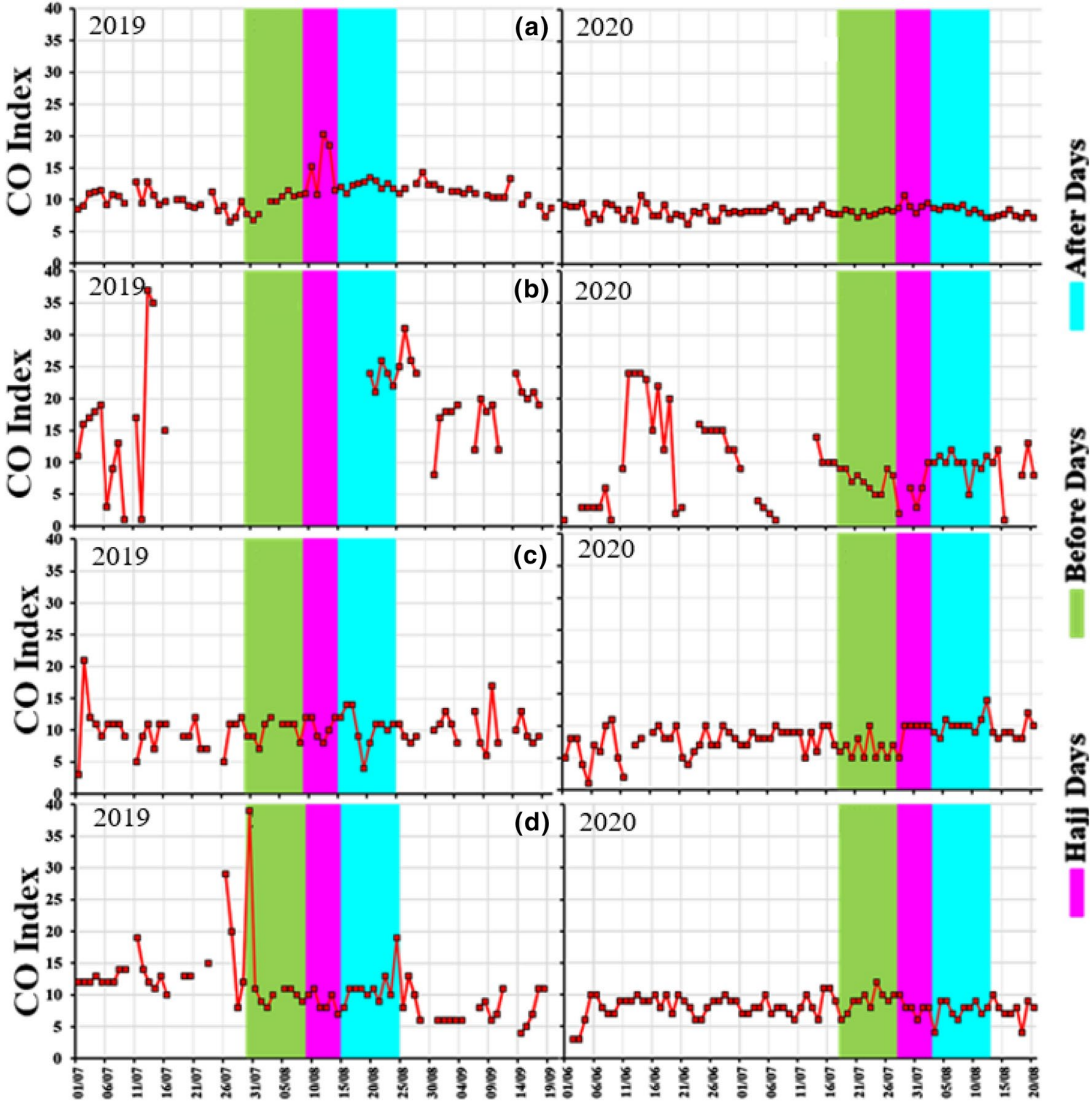
Temporal Variations of surface CO Index over **a** Mecca, **b** Jeddah, **c** Cradle of Gold Station, Madinah and **d** Badr Station, Madinah during 2019 and 2020

Temporal variations of CO Index are shown in (Fig. 10, Table 2) over Mecca, Jeddah, and Cradle of Gold and Badr stations near Madinah. At Mecca, in 2019, the average CO Index is 9.44, 14.54 and 12.30 [equivalent to 0.83, 1.28 and 1.08 ppm (parts per million)] before, during and after Hajj, respectively. In 2020, the average CO Index was 8.03, 9.17 and 8.50 (≡ 0.71, 0.81 and 0.75 ppm) before, during and after Hajj, respectively (Fig. 10a). A reduction of about 14% in CO during July and about 33% in August 2020 was observed. In Mecca (Fig. 10a), a pronounced increase in CO index (concentration) in 2019 Hajj time and reduction of about 28% during 2020 Hajj was observed due to restrictions associated with COVID-19 (before, during and after Hajj).

In Jeddah (Fig. 10b), there are only a few data points available during the 2019 Hajj period as data was missing for before and during Hajj periods. However, we observed higher CO Index values after the 2019 Hajj compared to 2020, where the average CO Index was 23.40 (equivalent to 2.06 ppm). The average CO Index is 7.30, 5.40 and 9.80 (equivalent to 0.64, 0.48 and 0.86 ppm) before, during and after 2020 Hajj, respectively (Fig. 10b).

We found a similar reduction in CO Index (concentration) at COD stations near Madinah. At COD (Fig. 10c), the average CO Index was 9.89, 10.50 and 10.40 (equivalent to 0.87, 0.92 and 0.92 ppm) before, during and after Hajj 2019. The average CO Index was 6.5, 9.17 and 10.20 (equivalent to 0.57, 0.81 and 0.90 ppm) before, during and after 2020 Hajj, respectively. At Badr station, (Fig. 10d), the average CO Index was 13.11, 9.00 and 11.30 (equivalent to 1.15, 0.79 and 0.99 ppm) before, during and after Hajj 2019. In 2020, the average CO Index was 9.00, 8.00 and 7.50 (equivalent to 0.79, 0.70 and 0.66 ppm) before, during and after Hajj, respectively. In 2020, we found higher in CO values 4 days before the Hajj and until the end of Hajj, an increase was also observed, a reduction in CO up to 25% during 2020 was observed compared to 2019.

**Fig. 11:**
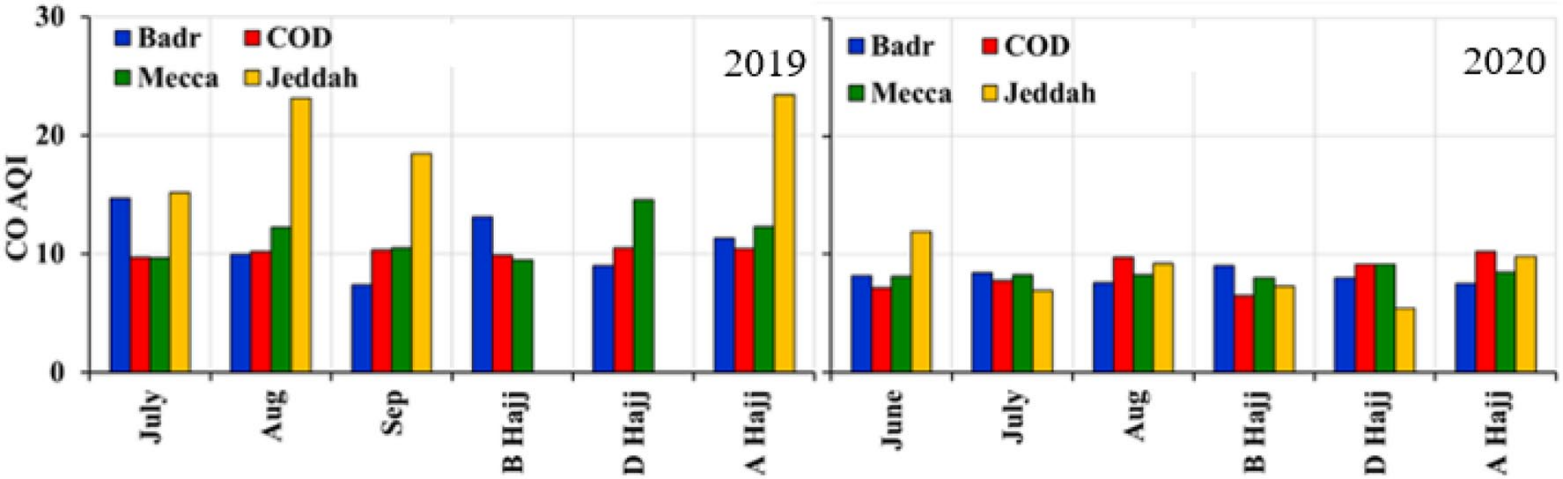
Average CO Index over Jeddah, Badr Station Madinah, COD (cradle of gold) Station, Madinah, and Mecca during 2019 and 2020. **B** Hajj (before Hajj); **D** Hajj (during Hajj) and **A** Hajj (after Hajj)

In Fig. 11, we have shown the average monthly variations of CO Index before, during and after Hajj. In 2019, the highest CO Index values were found in Jeddah, while a pronounced decrease in CO Index (concentration) during 2020 was observed compared to 2019 at all stations. In Mecca, the change in CO concentrations during Hajj was clearly observed during 2019 and 2020.

## Conclusions

Air quality in western parts of Saudi Arabia during the Hajj period was found to be affected mainly due to the pilgrims crowds, whereas the regional climatic conditions, mainly dust storms, also influence PM concentrations at a large scale. Air quality of the cities of Mecca, Jeddah, and Madinah in Saudi Arabia is affected by the annual multimillion human gathering during the time of Hajj.

Our detailed analysis shows that the air quality of Mecca city is highly affected by Hajj activities compared to the other two cities. The pronounced changes in air quality parameters clearly show the effect of pollutants associated with the large number of vehicles transporting pilgrims to Mecca before Hajj and activities along the road from the airport to Mecca. The pollution data also clearly show a reduction in pilgrims due to strict social gathering restrictions during the 2020 Hajj. Before Hajj, PM_10_, NO_2_, and CO concentrations decreased by 54.1, 44.7, and 14.4% in 2020 compared to 2019, respectively. During Hajj, NO_2_, and CO decreased by 54.7% and 36.7% respectively, while PM_10_ increased by 26.2%. After Hajj, NO_2_, and CO decreased by 64.7, 30.5%, respectively, while PM_10_ increased by 55.6%. The increase in PM_10_ during and after Hajj in 2020 is attributed to dust loading in the atmosphere as observed by Meteorological data.

In Jeddah, in 2019, no data were available before and during Hajj. After Hajj, 78.8, 58.2% declination in NO_2_, and CO, respectively, while a 63.4% increase in PM_10_ values were observed in 2020 compared to 2019. The NO_2_ and CO declination in Jeddah is another indicator of the effect of high traffic volume on anthropogenic emissions during Hajj.

On comparing 2019 and 2020, NO_2_ Index of various stations, we observed that NO_2_ Index was higher during 2019 at each station. Most of the pilgrims first arrived at Jeddah Airport and later they go to Mecca by road. Moreover, there are other anthropogenic sources in Jeddah, so the concentration was higher in Jeddah during 2019. During 2020, NO_2_ index values are comparable at both Mecca and Jeddah. Also, we found a higher NO_2_ Index during Hajj time in Mecca. Due to COVID-19 pandemic socio-economic restrictions, the anthropogenic emission was lower, overall, 2020 average values were lower compared to 2019.

In 2020, pilgrims were restricted to visit Madinah, so it was difficult to examine the effect of Hajj on air quality of Madinah. It was, however, noticed that during 2020 Hajj there were 70.3 and 73.8% reductions in NO_2_ values and 11.9 and 11.3% reductions in CO values compared to 2019 at both COD and Badr stations near Madinah. Such reductions may not be related to Hajj activities, but it could be related to the nationwide lockdown restrictions in Saudi Arabia during summer 2020. During and after Hajj, no significant changes in NO_2_ or CO were observed at the Badr station in 2020 compared to 2019, while a 77% declination was recorded in the COD station in 2020 compared to 2019.

Data shows that Jeddah has the highest values of NO_2_ and CO index in 2019 whereas a rapid reduction was observed in 2020. This shows that the air quality is not mainly affected by the holy gathering of the people in fact it is more due to the anthropogenic activities in the economic activity hub cities such as in Jeddah.

The results show that reducing the number of vehicles transporting pilgrims from Jeddah to Mecca and from Mecca to Madinah could help reducing air pollution during the annual Hajj season. Using environmentally friendly buses and trains to commute between the three cities could be a key solution to reduce the pollution effects. We also found that regional climatic conditions and dust storms play a significant role in PM_10_ variability over the Hajj cities. Even with a smaller number of pilgrims at the time Hajj, the average PM_10_ concentration is found to be higher in 2020.

Due to limited data, we are limited to show daily variations only for two years (as the data was available for 2019 and 2020). The result of 2020 will be benchmark for future study as the number of pilgrims are quite less in recent decades and these results will be helpful for future planning of Hajj in Mecca and Madinah.

## Data statement

All the data used in the present study are freely available, if needed we will provide data used in the present study to anyone.
